# Extracellular Mechanical Stimuli Alters the Metastatic Progression of Prostate Cancer Cells within 3D Tissue Matrix

**DOI:** 10.3390/bioengineering10111271

**Published:** 2023-10-31

**Authors:** Maggie Ditto, Diego Jacho, Kathryn M. Eisenmann, Eda Yildirim-Ayan

**Affiliations:** 1 Department of Bioengineering, College of Engineering, University of Toledo, Toledo, OH 43606, USA; 2 Department of Cell and Cancer Biology, College of Medicine and Life Sciences, University of Toledo Health Science Campus, Toledo, OH 43614, USA

**Keywords:** mechanical, stimuli, loading, prostate, cancer, bone, extracellular, actin, invasion, elongation, metastasis, static, cyclic, EQUicycler, cytoskeleton

## Abstract

This study aimed to understand extracellular mechanical stimuli’s effect on prostate cancer cells’ metastatic progression within a three-dimensional (3D) bone-like microenvironment. In this study, a mechanical loading platform, EQUicycler, has been employed to create physiologically relevant static and cyclic mechanical stimuli to a prostate cancer cell (PC-3)-embedded 3D tissue matrix. Three mechanical stimuli conditions were applied: control (no loading), cyclic (1% strain at 1 Hz), and static mechanical stimuli (1% strain). The changes in prostate cancer cells’ cytoskeletal reorganization, polarity (elongation index), proliferation, expression level of N-Cadherin (metastasis-associated gene), and migratory potential within the 3D collagen structures were assessed upon mechanical stimuli. The results have shown that static mechanical stimuli increased the metastasis progression factors, including cell elongation (*p* < 0.001), cellular F-actin accumulation (*p* < 0.001), actin polymerization (*p* < 0.001), N-Cadherin gene expression, and invasion capacity of PC-3 cells within a bone-like microenvironment compared to its cyclic and control loading counterparts. This study established a novel system for studying metastatic cancer cells within bone and enables the creation of biomimetic in vitro models for cancer research and mechanobiology.

## 1. Introduction

Prostate cancer is the second leading cause of death in males in the United States [1,2], where most prostate cancer-related deaths are due to bone metastases [3,4]. The five-year survival rate for prostate cancer bone metastasis patients is around 3%, while this rate is 56% for non-metastatic patients [2,5]. This emphasizes the need for an increased understanding of prostate cancer bone metastasis and the behavior of metastatic prostate cancer cells in the bone microenvironment [1].

The interaction between metastatic cancer cells and the bone microenvironment plays a crucial role in driving cellular and molecular changes in tumor cells to promote their migration within the bone [6,7]. Efforts have been put into investigating interactions of metastatic cancer cells with the bone microenvironment [8,9,10]. Primarily, animal models have been used in these investigations [11,12]; however, in vivo studies are associated with limited reproducibility, prohibitive cost, and difficulty in data interpretation due to the synergetic effects of multivariable factors [13]. As a result, physiologically relevant biomimetic three-dimensional (3D) in vitro models have been developed to overcome these limitations [2].

Biomimetic 3D in vitro models provide a cost-effective, reproducible, and well-defined platform to study the effects of multiple variables within the tumor/bone microenvironment on changes in metastatic cells [14,15,16]. In these models, cancer cells are seeded on mineralized or calcified bone tissue scaffolds resembling the native bone extracellular matrix (ECM). These 3D in vitro studies have primarily studied the role of bone-like microenvironments’ structure and/or composition on molecular and cellular changes in cancer cells [17,18]. For instance, Riechert et al. [13] used a mineralized and then decellularized matrix as a model to study prostate cancer cellular function in the bone microenvironment. Briefly, LNCap and PC-3 prostate cancer (CaP) cells were seeded onto a mineralized, ECM-like matrix to investigate the attachment, proliferation, gene expression profile, and MMP activity. Their results showed that when placed upon a mineralized matrix, CaP cells strongly adhered, proliferated, expressed markers consistent with a loss of epithelial phenotype, and showed increased invasive potential. In summary, they concluded that a mineralized matrix can be used to study tumor/bone microenvironment interaction. Thibaudeau et al. [19] utilized human primary osteoblast-derived matrices (OBM) to investigate the effects of bone matrix mimicking on the drug responsiveness of different cell lines. Although their results showed that OBM did not significantly affect cancer cells’ drug response compared to tissue culture plastic, their study introduced physiologically relevant bone matrix (OBM) for future bone metastasis studies. Pathi et al. [20] investigated the role of bone mineral hydroxyapatite (HA) in breast cancer bone metastasis. They cultured human MDA-MB-231 breast cancer cells onto the mineralized 3D HA scaffolds. Their results showed that tumor cell adhesion, proliferation, and the secretion of pro-osteoclastic cytokines were increased in mineralized tumor models as compared to non-mineralized tumor models. Based on these results, they concluded that breast cancer cell behavior is broadly affected by the presence of hydroxyapatite. Pathi et al. [21] has further investigated the effect of HA material properties, such as particle size and crystallinity, on metastatic breast cancer behavior. They seeded human MDA-MB231 breast cancer cells onto an HA scaffold with various particle sizes and crystallinity. Their results showed that smaller, poorly crystalline HA particles enhanced cancer cell adhesion and growth relative to larger, more crystalline HA particles, confirming HA particles’ role in bone metastasis. Sieh et al. [6] studied the interactions of osteoblast cells and CaP cells with polycaprolactone-tricalcium phosphate (mPCL-TCP) scaffolds by wrapping mPCL-TCP scaffold within the osteoblast sheets to create a 3D matrix for CaP cell culture. CaP cells were then seeded on this 3D matrix to study CaP metastasis in a bone-like microenvironment, showing that CaP cell-bone matrix interactions lead to elevated levels of matrix metalloproteinase (MMPs) and prostate-specific antigens (PSAs), which are all associated with CaP metastasis.

These 3D in vitro models shed light on the importance of cancer cell-bone microenvironments on cancer cell functions and offer potential tools for bone metastasis studies. However, without any exceptions, these studies and many others in the fields ignore the mechanical loading exerted on bone tissue due to the diurnal activities, and [22,23,24] increased constant pressure as a result of growing tumor [3,4]. For instance, metastasized cancer cells are exposed to a myriad of extracellular factors, including increased mechanical stimuli due to tumor growth within a confined space, matrix stiffness via stromal activation [5,6,7], interstitial fluid pressure due to ineffective vascularization, and production of soluble factors from cell-cell and cell-matrix interactions [1,25,26,27,28]. While the effects of matrix stiffness, interstitial fluid pressure, and soluble factors on cancer metastasis have been extensively studied [29,30,31], the influence of extracellular mechanical stimuli upon cancer cell migration within 3D matrix and establishment of new metastases remains relatively unexplored. Current 3D in vitro models can address such limitations by considering the mechanical extracellular factors in metastasis investigation and incorporating the cancer cells within the bone-like microenvironment.

In this study, we aim to create a physiologically relevant 3D in vitro system considering the mechanical stimuli affecting prostate cancer cells within the bone-like environment and the effect of these mechanical stimuli on driving metastatic spread within the bone. To create a biomimetic mechanically stimulated bone-like environment, two different types of mechanical loading conditions, static and cyclic mechanical loadings, were applied on 3D PC-3 embedded collagen constructs using a custom-built EQUicycler system. The static mechanical stimuli are reminiscent of the tumor-associated forces created on the cells within the bone-like environment, while the cyclic load condition represents the physical activity-associated forces created on the cells within the bone-like environment. From this work, we can create a 3D in vitro model to study the PC-3 cell-bone microenvironment in the presence of extracellular mechanical stimuli and to gain quantitative insight into how various extracellular mechanical stimuli affect the migration potential of PC-3 cells within the bone-like environment.

## 2. Materials and Methods

### 2.1. Preparation of Prostate Cancer Cell-Embedded 3D Collagen Tissue Matrix

Bone metastasis prostatic adenocarcinoma PC-3 cells (ATCC, Manassas, VA, USA) (not primary cells) were used in this study. The reason for choosing PC-3 cell lines over another popular cell line in a prostate cancer study, LNCap, was their origin difference. PC-3 is a bone-derived human prostate adenocarcinoma cell line, while LNCap is derived from lymph node metastasis and represents an early stage of prostate cancer [32,33,34,35]. In this study, we tried to identify the effect of mechanical stimuli within the bone microenvironment on bone metastatic cancer cells. We have used PC-3 cells rather than LNCap cell lines in this context.

**Figure 1:**
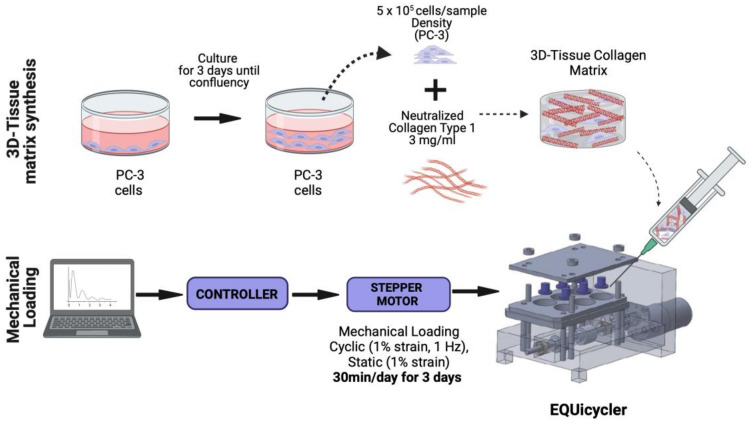
Schematic of the 3D tissue matrix synthesis and mechanical stimulation loadings applied. Created with Biorender.com.

PC-3 cells were populated and maintained in Kaighn’s Modification of Ham’s F-12 Medium (ATCC, Manassas, VA, USA) supplemented with 1% antibiotics (Life Technologies, Frederick, MD, USA) and 10% (*v*/*v*) fetal bovine serum (FBS) (Gibco, Waltham, MA USA) in a 37 °C, 5% CO_2_ environment. Rat-tail collagen type-I (BD Bioscience, San Jose, CA, USA) was used to create PC-3 cell-embedded 3D collagen constructs (Figure 1). Collagen type I solution was chosen to simulate the bone-like environment associated with PC-3 metastases because the bone organic matrix comprises approximately 90% collagen type I [36,37]. The organic matrix accounts for 35% of bone volume, so it is appropriate for use in a bone-environment scaffold [36].

Type I collagen solution was prepared based on manufacturer instructions. Briefly, the collagen stock solution was neutralized using phosphate-buffered saline (PBS) and 1 M NaOH to prepare neutralized collagen with 3 mg/mL concentration. After neutralization, PC-3 cells were mixed with collagen solution homogeneously with a concentration of 5 × 10^5^ cells/sample. Then, PC-3-embedded collagen solution was deposited around the deformable silicon post with a sample volume of 750 µL. After deposition, PC-3 cell-embedded samples were incubated overnight at 37 °C and 5% CO_2_ environmental conditions to allow cellular acclimation to the 3D collagen environment.

### 2.2. Mechanical Stimuli Application System to 3D PC-3-Collagen Tissue Matrix: The EQUicycler

The EQUicycler, an innovative, custom-built mechanical loading platform [14], was designed to create physiologically relevant mechanical stimuli for the PC-3 embedded collagen construct. Unlike other mechanical loading platforms, the EQUicycler system was designed to create predefined uniform mechanical strain on the 3D cell-embedded constructs without creating a gripping effect. The EQUicycler is designed to load the cell-embedded collagen construct to the platform through deposition around a deformable silicon post rather than using grips. The compression of the silicon post produces mechanical strain in the cell-embedded collagen ring, which surrounds the post. This way, we can create homogenous strain throughout the cell-embedded collagen construct without creating a gripping effect.

**Figure 2:**
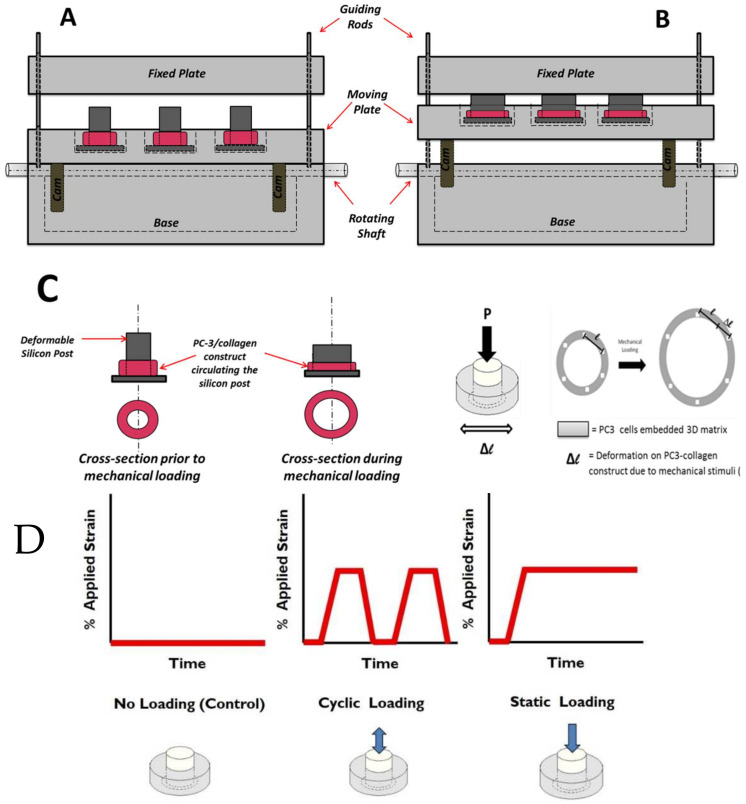
Schematic representation of EQUicycler system (**A**) before mechanical loading and (**B**) during the mechanical loading of the PC-3 embedded collagen construct. The displacement of the moving plate against the fixed plate causes compression of the silicon post, which creates mechanical strain on the cell-embedded collagen ring surrounding the post. (**C**) Schematic representation of the PC-3 cell-embedded collagen constructs before and during the mechanical loading. (**D**) Profile of applied strain during the mechanical loading associated with different experimental groups. “Design and Validation of Equiaxal Mechanical Strain Platform, EQUicycler, for 3D Tissue Engineered Constructs” BioMed Research International, Hindawi (2017) [11].

The EQUicycler’s working mechanism is based on creating a mechanical strain on the silicon post-hosting cell-embedded collagen 3D tissue matrix ring around it following our well-established protocol [14,15,22,24]. Figure 2 demonstrates the position of the moving plate and compression of the silicon posts before and during the mechanical loading.

In the EQUicycler system, the applied mechanical strain and its profile are dictated by the geometry of the cam mechanism. In the pear-shaped cam system, the pear-shaped cam remains motionless during rotation, and the moving plate becomes stationary for about half of a revolution. This period is called a dwell period. During the other half of the revolution of the cam, the moving plate rises and then falls. As the pear-shaped cam is symmetrical, the rise motion is the same as the fall motion. This profile creates cyclic displacement for the moving plate, which is utilized in the EQUicycler system to create cyclic reciprocating motion for the moving plate. The cam system is paused in a dwell period to create the static displacement.

This study applied three different mechanical loading conditions to the PC-3 cell-embedded collagen matrix using the EQUicycler system. These conditions are (1) control (no loading), (2) cyclic mechanical stimuli, and (3) static mechanical stimuli. Figure 2 demonstrates the profile of the applied strain during mechanical loading associated with different experimental groups.

Cyclic-loaded samples were subjected to 1% strain at 1 Hz for 30 min daily. Static-loaded samples received a constant 1% strain for 30 min daily. Control samples remained unloaded for the duration of the experiment. Loading was conducted for 3 days. All mechanical loading conditions utilized in this study are physiologically relevant. Bone mechanotransduction studies report that moderate activities (i.e., walking) create lower amplitude of mechanical stimuli within the bone with low frequencies (<2 Hz) thousands of times with an amplitude of 1% strain [33,36,37,38,39,40]. This study’s cyclic mechanical stimuli applied with 1% strain at 1 Hz represent the mechanical loading on the bone microenvironment due to physical activities. While cyclic mechanical stimuli represent the mechanical loading exerted on the bone microenvironment due to everyday activities, the static mechanical stimuli is an appropriate model for mechanical stimuli on cells within the bone environment due to body weight [40] and tumor growth [8,9,10].

### 2.3. Cytoskeletal Organization of PC-3 Cells in Response to Mechanical Stimuli within the 3D Tissue Matrix

The effect of mechanical loading upon cytoskeletal reorganization was assessed using confocal microscopy on days 0, 1, 2, and 3. For immunofluorescence, samples were washed in PBS and fixed with a 4% paraformaldehyde in PBS, permeabilized in a 0.2% Triton X-100/PBS, and incubated with Alexa Fluor 488-phalloidin (Invitrogen, Carlsbad CA, USA) for F-actin labeling and Alexa Fluor 594-DNase I (Invitrogen, Carlsbad CA, USA) for G-actin labeling. Samples were stained with a 4′,6-diamidino-2-phenylindole (DAPI) to visualize the cell nucleus. The samples were imaged using a Leica TCS SPE confocal microscope with 0.5 µm slice thickness. The number of slices for visualization was dependent on the cell size. Alexa Fluor 488-phalloidin was visualized using a 488 nm laser and an emission spectrum of 491 nm to 545 nm. Alexa Fluor 594-DNase I was visualized using a 568 nm laser and an emission spectrum of 590 nm to 685 nm. DAPI was visualized with a multiphoton laser tuned to 790 Hz. Samples were visualized using a 63× objective with oil and an optical zoom of 3.0 or 3.5× magnifications.

F-actin and G-actin associated with each cell were quantified using a histogram analysis provided by the GIMP2 image analysis software. Confocal images were exported as 8-bit images comprised of 255 red and green color intensities. A thresholding process was completed to remove background contamination from the lowest 10 color intensities during this analysis. Individual cell bodies were outlined using the Free Select tool within GIMP2 to complete this analysis. Following regional selection, the histogram tool was used to determine the total number of pixels of F-actin and G-actin for each selected area. The ratio of the values is given by the F-actin pixel count to the G-actin pixel count. For this analysis, three samples were prepared and analyzed for each experimental group, with *n* = 20 cells per sample. Samples were prepared independently of those utilized in cell elongation analysis. Samples were visualized using a 20× objective with water and an optical zoom of 1.8× magnification.

### 2.4. Changes in Cell Elongation Index of PC-3 Cells in Response to Mechanical Stimuli within the 3D Tissue Matrix

Cell viability was evaluated using a Live/Dead^®^ Assay (Invitrogen, Carlsbad CA, USA) per the manufacturer’s instructions, using a 1:4 ratio of calcein AM and ethidium homodimer 1. Briefly, 3D collagen–PC-3 cell constructs were removed from the mechanical loading platform and washed twice with PBS for 5 min. Following washing, collagen–cell constructs were incubated in Live/Dead assay solution for 30 min at 37 °C and 5% CO_2_. The samples were washed and fixed for 30 min in 4% paraformaldehyde/PBS. The samples were imaged using a Leica TCS SPE confocal microscope with multiphoton. Imaging was conducted with 2.5 µm slice thickness and 23–35 numbers of slices using a 10× objective with water and an optical zoom of 1.0× magnification. Calcein AM (staining cell cytoplasm) was visualized using a 488 nm laser and an emission spectrum of 491 nm to 545 nm. Ethidium homodimer-1 (dead cells) was visualized using a 568 nm laser and an emission spectrum of 590 nm to 685 nm.

The cell elongation index was calculated as the ratio of the long axis to the short axis of the cell based on a 3D rendering of serial optical slices collected by confocal microscopy. Individual cells within each image were analyzed using ImageJ (National Institute of Health, Bethesda, MD, USA) analysis software. At least three fields of view were selected from each image of each group for quantification. A cell elongation index of 1.0 represents a spheroidal cell, while an elongation index greater than 2.0 represents an elongated cell. Three samples per experimental group were analyzed for this analysis, with *n* = 50 cells per sample.

### 2.5. Proliferation of PC-3 Cells in Response to Mechanical Stimuli within the 3D Tissue Matrix

For cell proliferation analysis, 3D PC-3 cell constructs were subjected to static and cyclic mechanical loading, and the control group was removed from the mechanical loading platform on day 3. The proliferation was examined using a non-toxic alamarBlue (aB) assay (Biosource International, Carlsbad, CA, USA) for three days following the mechanical loading. For each group, four samples were used (*n* = 4). On the day of characterization, 10% (*v*/*v*) of aB assay solution was added to each sample and incubated for 4 h at incubator conditions. Following the incubation, a 250 µL solution of the color product was taken out from the wells and put into a 24 well-plate to measure the fluorescence intensity using a microplate fluorometer (Wallac 1420, Ramsey, MN, USA) at 545 nm excitation and 585 nm emission wavelengths.

### 2.6. Changes in Invasion Capacity of PC-3 Cells in Response to Mechanical Stimuli within the 3D Tissue Matrix

**Figure 3:**
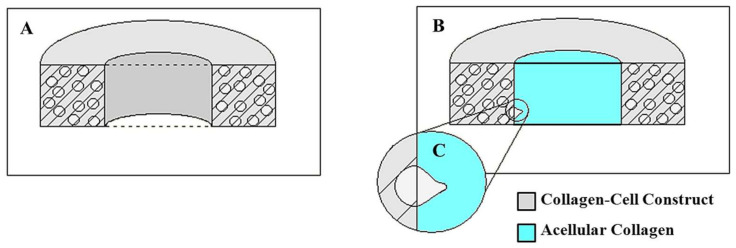
(**A**) The longitudinal section of the PC3-embedded collagen construct matrix (**B**) The longitudinal section of the invasion model assay (collagen matrix and acellular insert) (**C**) Schematic representation of penetrating PC3 cells into acellular collagen insert (invasion model).

A custom-built invasion assay was used to understand the changes in the invasion capacity of prostate cancer cells within the 3D bone-like environment upon mechanical stimuli. In this assay, we investigated the invasion of PC-3 cells within a mechanically loaded bone-like environment into the adjacent acellular collagen construct. Figure 3 displays the schematic representation of the PC3-embedded construct and the penetration of PC3 cells from this construct to an acellular 3D matrix.

Briefly, following the 3-day mechanical loading as described in Section 2.1 and Section 2.2, PC-3 embedded constructs were removed from EQUicycler, and 500 μL acellular collagen solution with a concentration of 3 mg/mL was deposited into the center of each PC-3 embedded circular construct (Figure 3A). The constructs were then incubated for t = 0, 12, and 24 h. Each time, the PC-3 cell-embedded and acellular 3D construct were fixed with 4% paraformaldehyde for 1 h and stained with Alexa Fluor 488 conjugated with phalloidin (Life Technologies, Frederick, MD, USA) to tag the cells. Then, the constructs were cut transversely to expose the center of the PC3/collagen matrix and acellular collagen inserts for visualization under a Leica Confocal microscope with a 488 nm laser and an emission spectrum of 491 nm to 545 nm.

### 2.7. Changes in Gene Expression of PC-3 Cells in Response to Mechanical Stimuli within the 3D Tissue Matrix

To identify the effect of mechanical stimuli on changes in the molecular level, the expression level of N-Cadherin (N-Cad) was evaluated. N-Cad was chosen because the increased expression of N-Cad is associated with increased invasiveness of metastatic potential of cancer cells [41,42,43,44].

A quantitative real-time polymerase chain reaction (RT-qPCR) was used to analyze the relative gene expression of N-Cad. Briefly, the 3D tissue matrices were mechanically disrupted on characterization day, and then the RNA was extracted using TRIzol reagent (TermoFisher, Carlsbad, CA, USA). According to the manufacturer’s instructions, the extracted RNA was reverse transcribed into cDNA using the Superscript IV kit (Invitrogen, Carlsbad CA, USA). Then, RT-PCR was carried out using TaqMan SYBR in the iCycler IQ detection system from Bio-Rad. Using the ∆∆Ct method, it was possible to determine the relative gene expression for the fold difference between the control and the mechanically loaded groups. Glyceraldehyde-3-phosphate dehydrogenase (GAPDH) was the housekeeping normalization gene in the ∆∆Ct method. The primer sequences for the genes were designed using NCBI primer blast software and synthesized by Integrated DNA Technologies (IDT, Coralville, IA, USA). N-Cadherin forward-5′ TGGAGAACCCCATTGACATT ′3 and reverse-5′ TGATCCCTCAGGAACTGTCC ′3 and GAPDH forward-5′ AGAAGGCTGGGGCTCATTTG ′3 and reverse-5′ AGGGGCCATCCACAGTCTTC ′3.

### 2.8. Statistical Methods

The cell elongation index and actin polymerization ratio were analyzed to determine the significance of the effect of the mechanical loading regime on cell phenotypic behavior. Data populations were first analyzed for equivalent variance. The cell elongation index and actin polymerization ratio exhibited populations with unequal variance. Populations were then analyzed for individual data distribution. This analysis returned a non-normal sample distribution for each analyzed data set. Finally, the significance of the mechanical loading regime no cell phenotype was analyzed using Mood’s median test. This test was chosen for its robust resistance to differing variance and distribution types between data sets, as well as its resistance to population outliers. This analysis initialized a 95% confidence interval.

## 3. Results

### 3.1. Mechanical Stimuli within the 3D Tissue Matrix Mediate Cytoskeletal Reorganization of PC-3 Cells

**Figure 4:**
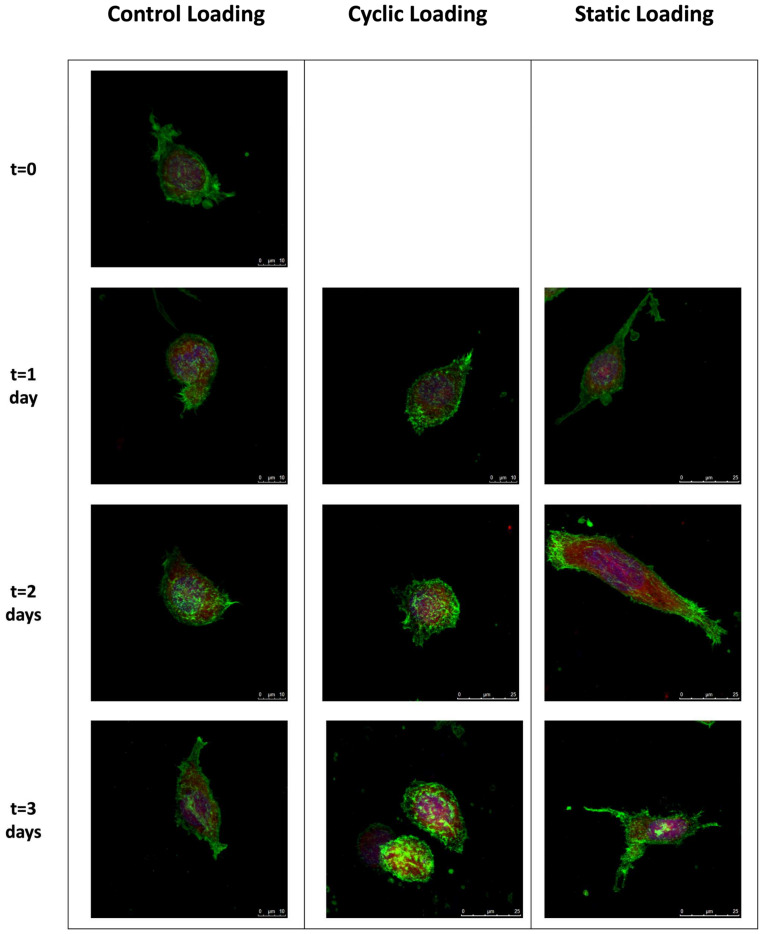
The confocal images demonstrate the changes in the cytoskeleton organization of PC-3 cells under static, cyclic, and no mechanical stimuli conditions over three days. Green: F-actin, red: G-actin, blue: nucleus. Images were taken with 63× objective. For day 0, only one representative image is shown, as no mechanical stimulation was applied. Scale bar = 10 µm for the control group (day 1,2,3) and cyclic loading (day 1) and 25 µm for cyclic and static mechanical stimuli group (day 1,2,3).

Confocal microscopy assessed the changes in the cytoskeletal reorganization of PC-3 cells within the collagen type I matrix before and after the cyclic and static mechanical stimulation on days 0, 1, 2, and 3 (Figure 4). F-actin (green) and G-actin (red) were visualized to investigate cytoskeletal reorganization in response to mechanical stimuli at each time point. The cells’ nuclei (blue) were also visualized to observe the possible migratory tendencies in response to mechanical stimuli.

We observed differential effects upon PC-3 cell morphologies within the collagen constructs following the control, static, or cyclical loading conditions. In control samples, where no loading was applied, a characteristic amoeboid morphology of PC-3 cells and a centrally located nucleus were observed. Upon 2 and 3 days of control conditions, cells assumed a mild spindle shape without dramatically altering the length of their long axis. Similar morphologies and nuclear localizations were observed in PC-3 cells within the collagen constructs where cyclic loads were applied for 1–3 days. However, on day 3, PC-3 cells in the cyclic experimental group showed an increased F-actin accumulation at the cell membrane, indicating the induction of cytoskeletal reorganization. PC-3 cells in the static loading group revealed dramatic changes in cell shape and cytoskeletal reorganization. Relative to their cyclic and control group counterparts, static loading for 1–3 days induces a dramatic cellular elongation accompanied by a polarized nucleus. This behavior suggests the ability of static mechanical stimuli within the bone-like environment to increase the migratory tendencies of PC-3 cells, potentially through induction of cytoskeletal reorganization and polarized cell elongation.

### 3.2. Mechanical Stimuli Mediate PC-3 Cell Morphology within the 3D Tissue Matrix

**Figure 5:**
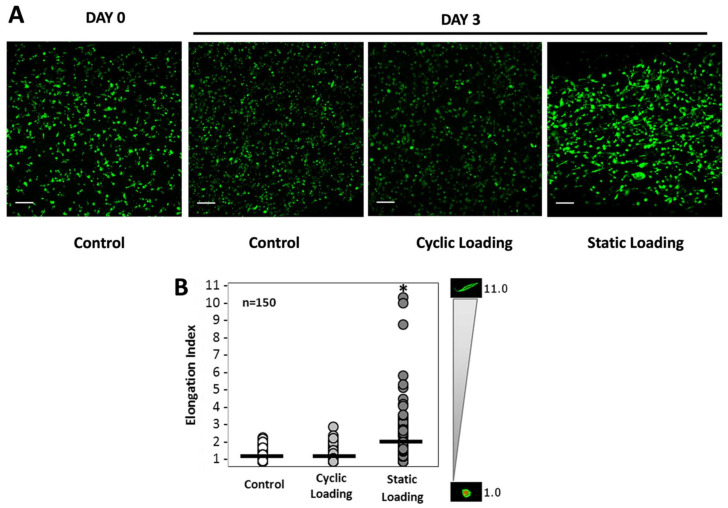
(**A**) Confocal images of PC-3 cells embedded within the 3D collagen matrix following the mechanical stimuli. The green color is the cell cytoplasm. The scale bar is 50 µm. (**B**) Cell elongation index (EI) analysis for PC-3 cells within the 3D collagen matrix at day 3. The EI is calculated by dividing the cell’s long axis by the cell’s short axis, where EI = 1 designates an amoeboid cell, and EI ≥ 2 is designated as an elongated cell. Mean represented by the black line. The gradient scale to the right designates the spectrum of cell measurements with corresponding representative images of cell morphologies. * *p* < 0.001 relative to unloaded control. At least 3 fields of view from a set of images were selected from each group for quantification (*n* = 3).

We then quantitatively assessed the impact of the various mechanical stimuli on cellular morphologies by measuring the cell elongation index (EI). The EI is calculated by dividing the cell’s long axis by the cell’s short axis, where EI = 1 designates an amoeboid cell, and EI ≥ 2 is designated as an elongated cell. Mechanical stimuli of varying conditions (control, cyclic, and static) were applied to PC-3 cells embedded in collagen constructs, and the cell elongation index was calculated in the fixed cells. Figure 5A shows immunofluorescent images of cells within the collagen matrix under variable mechanical stimuli conditions, with a pronounced elongated morphology present upon 3 days of static loading, relative to the more rounded morphologies in control and cyclic-loaded cells. Individual cell length and height measurements were then collected, and elongation indices were calculated for at least 150 cells after 3 days of loading (Figure 5B).

Additionally, Figure 5B shows that the mean elongation value of samples under static loading (2.50) was greater than those subjected to cyclic loading (1.35) or no loading (1.30). Statistical analysis revealed that only applied static loading significantly increased cell elongation compared to unloaded and cyclic-loaded constructs (*p* < 0.001).

### 3.3. Altered Actin Dynamics in PC-3 Cells within the 3D Tissue Matrix Are Dependent on the Type of Mechanical Stimuli

**Figure 6:**
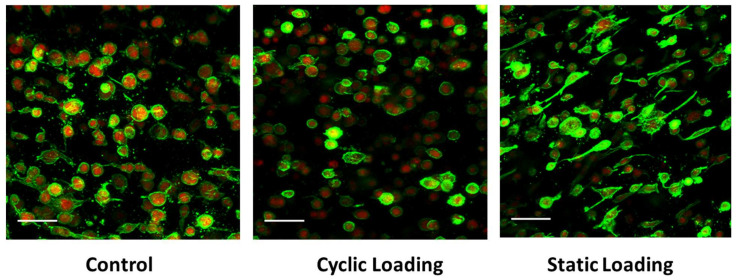
F-actin (green) and G-actin (red) presence in PC-3 cells within the 3D collagen matrix under control (unloaded), cyclic, and static loading conditions. Scale bar = 25 µm.

The increased metastatic potential of cancer cells can be affected through changes in the F-actin dynamics [45,46,47,48]. To understand changes in PC-3 cell F-actin dynamics in response to various mechanical stimuli within the bone-like microenvironment, we quantified the ratio of polymerized F-actin (green) to monomeric G-actin (red) by immunofluorescence (Figure 6). Within the bone-like environment, relative to control and cyclic loaded cells, the amount of F-actin increased on PC-3 cells under static mechanical stimuli, suggesting the increased migratory potential of these cells.

**Figure 7:**
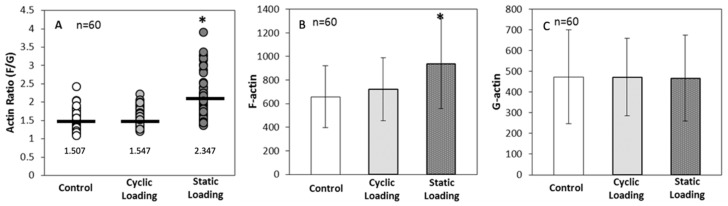
(**A**) Single-cell actin polymerization analysis for actin ratio (F/G) value plot per experimental group (mean represented by black line). Ratios were not standardized to any group * *p* < 0.001 relative to control. Actin polymerization analysis for (**B**) cellular F-actin levels within each sample group and (**C**) cellular G-actin levels within each sample group. The error bar represents mean ± SD (*n* = 60). * *p* < 0.001 relative to control.

F-actin levels were then measured on Day 3 of loading, and the degree of actin polymerization was calculated numerically as the ratio of F-actin (green) intensity to G-actin (red) intensity. Indeed, applied static loading significantly increased F-actin polymerization within the cell, compared to cyclic loading and control (unloaded) conditions (*p* < 0.001) (Figure 7). These data then further raised the question of whether the increase in polymerization was related to a significant increase in the total cellular pool of F-actin or a significant decrease in G-actin. For this, the ratio was calculated by comparing each cell’s F- and G-actin intensities, keeping the number of cells analyzed consistent (*n* = 60 across all groups).

Data in Figure 7 suggest that static mechanical stimuli within the bone-like environment significantly increased polymerized F-actin compared to both cyclic and control groups (*p* < 0.001). On the other hand, monomeric G-actin did not significantly change in response to any form of mechanical stimuli (*p* = 1.000). These data indicate that the increase in the F/G-actin ratio (2.347 in static loading) resulted from an increase in F-actin within the cells.

### 3.4. Proliferation of PC-3 Cells in Response to Mechanical Stimuli

**Figure 8:**
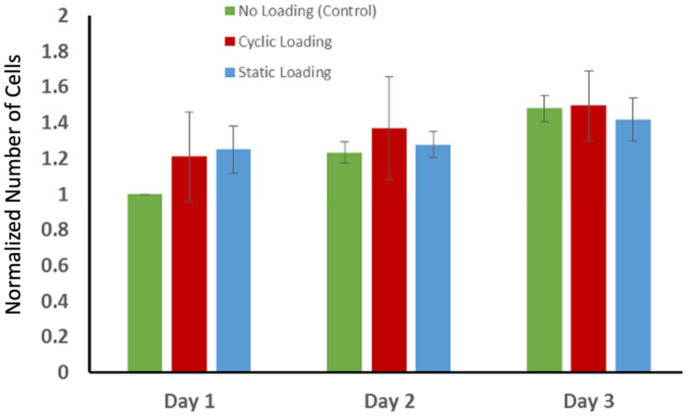
The changes in the number of cells over three days following the mechanical stimuli. The data on the number of cells on each characterization day were normalized with respect to the number of cells on day 1 to track the possible cell growth. Each bar represents the mean ± SD of the normalized number of cells for experimental groups (*n* = 4).

The changes in actin orientation and polymerization through mechanical stimuli raised another question: whether the proliferation of the PC-3 cells changed in response to mechanical stimuli. To answer this question, the proliferation of the PC-3 cells within collagen was examined using a non-toxic alamarBlue (aB) assay for three days following the mechanical loading. Figure 8 demonstrates the changes in the number of cells over three days following the mechanical stimuli. The number of cells slightly increased for each experimental group over three days. However, there was no statistically significant difference in proliferation of the PC-3 cells between experimental groups. The cell proliferation graph demonstrates that static and cyclic mechanical stimuli applied on the PC-3 embedded collagen construct do not alter the proliferation capacity of PC-3 cells.

### 3.5. The Changes in Expression of Metastasis-Associated Genes in Response to Mechanical Stimuli

**Figure 9:**
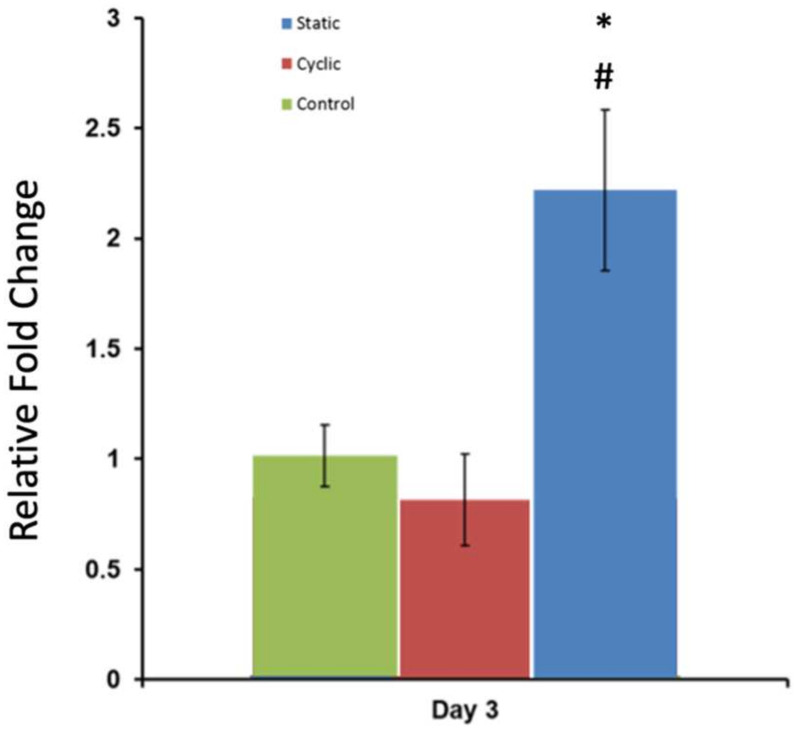
Normalized fold change in N-Cad gene expression following three-day static, cyclic, and no mechanical stimuli (control). GAPDH was used as the housekeeping gene. Values are shown as the mean ± SE (*n* = 3). * *p* < 0.001 relative to control, # *p* < 0.001 relative to experimental group.

To identify the effect of mechanical stimuli on changes in the molecular level, the expression level of N-Cadherin (N-Cad) was evaluated using real-time quantitative polymerase chain reaction (RT-PCR). The normalized fold change in the gene expression level of N-Cad with respect to the housekeeping gene (GAPDH) is given in Figure 9. The statistical analyses for the results were conducted using the *t*-test for two samples assuming equal variances from the Data Analysis ToolPak. Gene expression of N-Cadherin showed a two-fold increase in the static mechanical stimuli group, while cyclic and control groups showed no fold change greater than one in N-Cad expression (*p* > 0.05).

### 3.6. Mechanical Stimuli Mediate Invasion Capacity of PC-3 Cells to the Adjacent Environment

**Figure 10:**
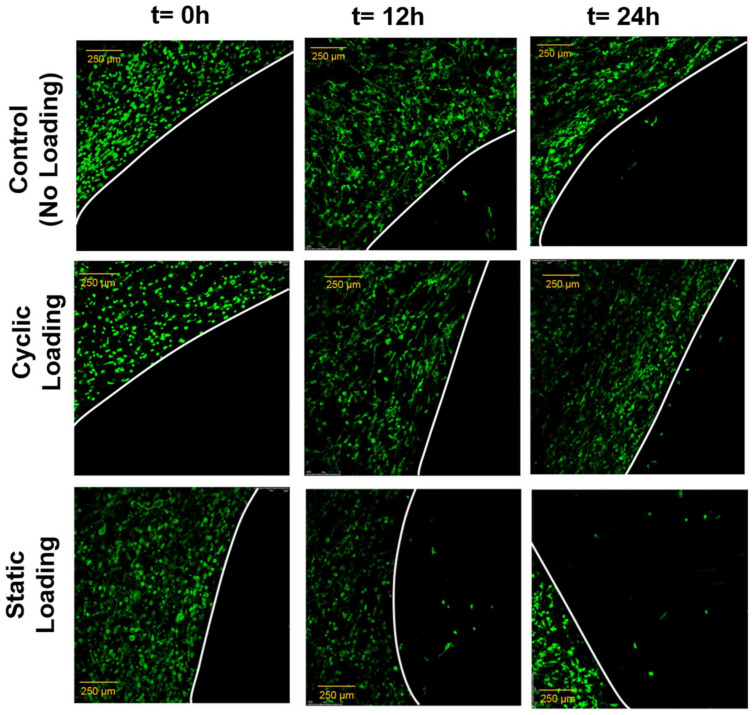
Immunofluorescence images of PC-3 cells (green) at t = 0 h, t = 12 h, and t = 24 h after 3 days of mechanical stimuli. The images demonstrate that the number of PC-3 cells invaded into the acellular construct and their longest distance translocation into the acellular construct increased in the static loading group (static loading group t = 24 h). The white line is a hypothetical line representing the border between cellular and acellular constructs. Scale bar 250 µm.

The changes in invasion capacity of PC-3 cells through the 3D matrix as a function of mechanical stimuli were assessed using a custom-built invasion assay. Figure 10 demonstrates the invasion assay results. The representative immunofluorescent images of PC-3 cells (green) within the collagen matrix and the invasion of those to the acellular collagen matrix over the predefined time interval are given for each experimental group.

Immunofluorescent images of PC-3 cells invaded the acellular collagen construct and demonstrated that static mechanical stimuli increased the invasion capacity of PC-3 compared to the control and cyclic mechanical stimuli. At 12 and 24 h after 3 days of mechanical stimuli, static loading maintained the greatest number of invaded cells within the acellular construct, with the longest distance translocation from the borders. PC-3 cells exposed to cyclic mechanical stimuli did not invade the acellular collagen after 12 h and showed slight invasion at 24 h. In the control group (no mechanical stimuli), the invasion assay results were interesting because, at 12 h, the PC-3 cells demonstrated similar invasion capacity with the static mechanical stimuli group. However, the trend slowed down at 24 h.

## 4. Discussion

With the growing acceptance of tumor microenvironment-induced changes in morphology and the invasive phenotype of cancer cells, more in vitro models have emerged to capture the tumor/host tissue environment and their interactions [26,49,50,51,52,53]. However, current 3D in vitro bone metastasis models often neglect the extracellular mechanical stimuli acting on cancer cells. By integrating biomimetic design and tissue engineering strategies, we have developed a novel 3D cell-embedded system to investigate the pro-metastatic role of mechanical stimuli within a 3D bone-like environment. The results of our study indicate that static mechanical stimuli within the bone-like 3D environment mediate the invasive potential of PC-3 cells through altering actin organization and polymerization.

The coordinated polymerization and reorganization of the actin cytoskeleton is crucial for tumor cell motility [7,54,55,56]. As metastatic potential increases, actin dynamics are altered, driving the progression of malignant cells to a more invasive phenotype [57,58]. To investigate the effect of mechanical stimuli on these phenotypic indicators of metastasis, we analyzed at a single cell level F-actin cytoskeletal alterations daily in response to mechanical loading (Figure 6). As the complete loading regime spanned 3 days, samples were analyzed on days 0–3. The actin cytoskeleton was evaluated using multiple parameters, including cell elongation, F-actin reorganization, and alterations to cell directional behaviors, as indicated by changes in nuclear polarity. While control and cyclic-loaded PC-3 cells embedded in collagen constructs maintained an amoeboid or mildly elongated morphology through 3 days of loading, static-loaded cells revealed a shift to a dramatic and sustained elongated morphology by day 1. This elongated morphology was sustained through 3 days of loading, with focal F-actin accumulation at the cell surface and polarized nuclear localization (Figure 6).

Concurrent with these changes, elongation indices and quantification of F/G-actin ratios indicated increases in F-actin accumulation and elongation indices only upon static loading (Figure 7). Previous studies on the association of cell elongation and actin polymerization showed a direct correlation between increased cell elongation and actin polymerization with increased cell metastatic potential [59,60]. These studies demonstrated that metastatic cell bodies became elongated and polarized compared to the non-metastatic cells within the tumor body. Thus, we have visualized the morphology of PC-3 cells and quantified the elongation within a bone-like environment under various mechanical stimuli. Analysis of the confocal images (Figure 5A) and elongation index (Figure 5B) revealed that only static mechanical loading significantly increased cell elongation compared to both cyclic and control conditions (*p* < 0.001).

The cell elongation is also associated with the dynamic reorganization of the actin cytoskeleton [23,46,47,48]. The polymerized actin amount (F-actin) in malignant cells increases with increasing metastatic potential [45]. Thus, changes in actin polymerization were observed in response to mechanical stimuli within a bone-like environment. Actin polymerization was calculated via intensity analysis of immunofluorescent images (Figure 5 and Figure 6). The actin polymerization data suggested that static mechanical stimuli significantly increased the actin polymerization and the F/G ratio (Figure 7) through increasing polymerized F-actin within the cells (Figure 7). The reduction in the expression of F-actin in the sample subjected to cyclic loading can be attributed to the fundamental differences in the nature of the stimulation compared to the other group, which experienced constant static stimulation. This distinction in the mechanical stimulus likely played a pivotal role in the alteration of F-actin expression [61,62].

These data indicate that static mechanical stimuli of PC-3 cells within the 3D bone-like construct, versus cyclic or control (no mechanical stimuli), drive changes in the F-actin cytoskeleton consistent with inducing a more metastatic phenotype. This fact can be explained by the strength of the physical connection between mechanically loaded extracellular matrix (ECM) and integrin. The effect of extracellular stimuli, including mechanical loading, is transferred to the intracellular space by the integrins, a large family of cell-surface receptors that bind ECM components, organize the cytoskeleton, and activate intercellular signaling pathways. It has been proven that mechanical forces typically upregulate integrin, reinforce integrin–ECM linkage, and increase motility and invasion [8,9,10,63,64]. The continuously applied mechanical loading (static loading) may alter the strength of integrin-ECM binding, which, in turn, would help integrin to transfer the mechanical stimuli effectively to the intracellular components. Subsequently, this interaction would change cytoskeleton organization and actin filament formation.

The effect of mechanical stimuli on proliferation and metastasis-associated gene expression of PC-3 cells within bone-like environments has also been studied. The cell proliferation result indicated that proliferation did not reveal any definitive difference between control and mechanically loaded samples (Figure 8). The gene expression data from RT-PCR (Figure 9) demonstrated that there is more than a two-fold change increase in the gene expression level of N-Cad in the static mechanical stimuli group, while cyclic and control groups did not show any fold change in N-Cad expression. The proliferation and gene expression can be attributed to the magnitude of mechanical strain applied in this study. Metastasis is a multistep process that includes changes first in cell morphology through actin polymerization/remodeling and the subsequent coordinated molecular changes. The applied strain and mechanical stimuli duration utilized in this study (1% strain, 30 min/daily) might have been ample for initiating the possible metastasis but not enough for changing the entire molecular blueprint of the PC-3 cells for a complete phenotypic shift towards metastasis. Further studies can be expanded with prolonged mechanical stimuli exposure and increased strain values to identify what mechanical stimuli trigger morphological and molecular changes in metastatic cancer cells. A custom-built invasion assay has been used to understand whether metastasis/invasion has been physically initiated. The invasion of PC-3 cells to the 3D acellular collagen matrix has been visualized over 12 and 24 h following the mechanical stimuli (Figure 10). The data demonstrated that static mechanical stimuli promoted the invasion of PC-3 cells. The samples strained with static mechanical stimuli maintained the greatest number of invaded cells within the acellular construct with the longest distance translocation from the border of the cellular/acellular construct (Figure 10).

Overall, this study provides a biomimetic and physiologically relevant 3D in vitro system to study the effect of extracellular mechanical stimuli on driving metastatic spread within a 3D bone-like environment. The results suggest that static mechanical stimuli applied with 1% strain for 30 min/daily for three days trigger the cytoskeletal reorganization, cell polarization, and invasion capacity of PC-3 cells toward metastasis. We hope this study will lead to new investigations considering extracellular mechanical factors in understanding metastasis and disease progression within the bone.

## 5. Conclusions

While some efforts have been made toward understanding the effects of bone extracellular matrix components on cancer cells’ functions and disease states, none of these studies have considered mechanical stimuli applied from extracellular space to the cancer cell-embedded bone microenvironment. In this work, to create a biomimetic mechanically stimulated bone-like environment, two different types of mechanical loading conditions, static and cyclic mechanical loadings, were applied on 3D PC-3 embedded collagen tissue matrices using an innovative, custom-built EQUicycler system. Our results suggest that static mechanical stimuli (1% strain for 30 min/day) applied to the PC-3 embedded bone-like environment change prostate cancer cells’ cytoskeletal reorganization, polarity (elongation index), expression level of metastasis-associated gene, and invasion potential within the 3D bone-like microenvironment. Thus, studies focusing on bone/metastatic cancer interfaces must consider extracellular mechanical stimuli.

## Data Availability

The data sets generated during the current study are available from the corresponding author upon reasonable request.
